# 

**Published:** 1890-11

**Authors:** 


### CONTENTS.

### ORIGINAL COMMUNICATIONS—

Opening Address of the Atlanta Medical College, Oct. 1st, 1890. By Prof. H. L. Jones ..... 515

Prof. Flint's Doctrine of the Self-Limitation of Phthisis. By Wm. Porter, M. D. ..... 523

A Case of Puerperal Convulsions, Four Days After Delivery. By Dr. V. O. Lockhart, Homer, Ga ..... 532

### CORRESPONDENCE—

Our New York Letter ..... 534

### SOCIETY REPORTS—

Mississippi Valley Medical Association ..... 539

Atlanta Society of Medicine ..... 554-557

### BOOK REVIEWS—

Annual of the Universal Medical Sciences ..... 564

Diseases of the Rectum and Anus ..... 565

Scheme of the Antiseptic Method of Womb Treatment ..... 565

### EDITORIAL—

The Southern Surgical and Gynecological Association ..... 567

The Responsibilities of Physicians who Attend Patients Afflicted with Contagious or Infectious Diseases ..... 569

Items ..... 570-577

Index to Advertisements on Page xix.

Reading Notices on Page xviii.

Premium List on Pages xxxiv and xxxv.

